# Impact of Glucocorticoids on Immune Checkpoint Inhibitor Efficacy and Circulating Biomarkers in Non–Small Cell Lung Cancer Patients

**DOI:** 10.1158/2767-9764.CRC-25-0051

**Published:** 2025-07-07

**Authors:** Lauren Polyakov, Angelina Lim, Alexandra Meyer, Aubree Mades, Joshua Ni, Ryan Cooper, Shirley Ye, Ryutaro Kajihara, Takaaki Oba, Leslie Contreras, Eihab Abdelfatah, Joy Sarkar, Junko Matsuzaki, Ming Li, Rajeev Sharma, Brahm H. Segal, Robert C. Hsu, Hongbin Chen, Jorge Nieva, Fumito Ito

**Affiliations:** 1 Department of Surgery, University of Southern California, Norris Comprehensive Cancer Center, Los Angeles, California.; 2 Department of Medicine, University of Southern California, Norris Comprehensive Cancer Center, Los Angeles, California.; 3 Center for Immunotherapy, Roswell Park Comprehensive Cancer Center, Buffalo, New York.; 4 Department of Hematology and Immunology, Faculty of Life Sciences, Kumamoto University, Kumamoto, Japan.; 5 Division of Breast and Endocrine Surgery, Department of Surgery, Shinshu University School of Medicine, Matsumoto, Japan.; 6 Department of Surgical Oncology, Roswell Park Comprehensive Cancer Center, Buffalo, New York.; 7 Department of Population and Public Health Sciences, University of Southern California, Norris Comprehensive Cancer Center, Los Angeles, California.; 8 Department of Medicine, University at Buffalo Jacobs School of Medicine and Biomedical Sciences, The State University of New York, Buffalo, New York.; 9 Division of Endocrinology, Hackensack Meridian Hackensack University Medical Center, Hackensack, New Jersey.; 10 Department of Medical Oncology, Roswell Park Comprehensive Cancer Center, Buffalo, New York.; 11 Department of Molecular Microbiology and Immunology, University of Southern California, Norris Comprehensive Cancer Center, Los Angeles, California.

## Abstract

Corticosteroids are frequently prescribed to patients with non–small cell lung cancer (NSCLC) for palliation of cancer-related symptoms; however, the potential impact of baseline steroid use on immune checkpoint inhibitor (ICI) therapy and its underlying mechanisms remain unclear. In this study, we evaluated clinical outcomes of 277 patients with NSCLC treated with ICI therapy at two academic institutions. Twenty-one patients (8%) were taking steroids at the start of ICIs. Patients on baseline steroids had a lower overall response rate with markedly shorter progression-free survival and overall survival compared with those not receiving steroids. In multivariate analysis, steroid use was the only significant independent risk factor for disease progression and mortality in both independent cohorts, Roswell Park Comprehensive Cancer Center (*n* = 88) and University of Southern California (*n* = 189). A baseline peripheral blood neutrophil-to-lymphocyte ratio <5 was a strong prognostic indicator; however, the prognostic value of neutrophil-to-lymphocyte ratio was absent in patients receiving steroids. Additionally, the baseline frequency of circulating CX3CR1^+^CD8^+^ T cells was substantially lower in patients on steroids. Using a bedside-to-bench approach, we found that concurrent steroid use significantly decreased antitumor efficacy of anti–PD-1 therapy and attenuated the increase of CX3CR1^+^CD8^+^ T cells in mice bearing MC38 tumors whereas discontinuation of steroid at the start of treatment did not make a negative impact on survival. Collectively, baseline steroid use was associated with worse outcomes and decreased frequency of circulating differentiated effector T cells in patients with NSCLC. Caution should be taken when interpreting the results from circulating immune-related biomarkers in patients on steroids.

**Significance:**

The impact of corticosteroids, widely prescribed for palliation of cancer-related symptoms, on ICI therapy remains unclear. This study shows that baseline steroid use is a negative independent prognostic factor in patients with NSCLC undergoing ICI therapy and provides insights into the decreased T-cell effector differentiation and utility of predictive blood-based markers by steroids.

## Introduction

Immune checkpoint inhibitor (ICI) therapy has been revolutionary for patients with non–small cell lung cancer (NSCLC), with anti–PD-1/PD-L1 regimens becoming a cornerstone of treatment (1–3). Improved clinical outcomes with the addition of chemotherapy to ICI therapy in patients with negative to low PD-L1 expression in the tumor further expanded the indication for these regimens (4–6). However, intense research is ongoing to understand mechanisms of resistance to ICI therapy as only a subset of patients receive durable clinical benefits. Multiple prognostic factors, such as performance status, smoking history, tumor histology, and/or the presence or absence of brain metastases, have been shown to have some predictive value for response to ICIs (7–14).

With the continued success of ICIs, many studies have sought ways to enhance their benefits while minimizing shortcomings. One key finding is the potential harmful interaction between ICIs and steroid use. Steroids have historically been used to manage immune-related adverse events (irAE) from ICI therapy (15, 16) and their use for the treatment of irAEs does not seem to interfere with clinical benefits, possibly because the presence of irAEs is often associated with favorable response to immunotherapy (11, 16–20). In contrast, baseline steroid use has been associated with decreased response rates and poor prognosis in multiple studies (7–13, 21). Despite known negative consequences, steroids are still widely prescribed for palliation of cancer-related symptoms, such as brain metastases, anorexia, or respiratory disease. However, it remains unclear whether reducing or tapering steroids prior to initiation of ICI therapy could decrease the negative impact of steroids on treatment outcomes (7). Furthermore, underlying immunomodulatory mechanisms of steroids in the context of ICI therapy are not fully understood.

Biomarkers are another ongoing topic of research in ICI therapy for NSCLC (22). Although tumor PD-L1 expression guides therapeutic decisions, some patients with PD-L1–negative tumors respond to anti–PD-1 monotherapy, highlighting the challenge of using a single biomarker to predict efficacy (23, 24). Moreover, no biomarkers currently predict the benefit of adding chemotherapy to anti–PD-1 therapy in chemo-immunotherapy regimens. This underscores the need for novel predictive biomarkers, especially blood-based ones, to optimize ICI therapy for patients with NSCLC. Emerging evidence suggests that neutrophils, a dominant immune cell type in NSCLC, inhibit the immune system by suppressing the cytolytic activity of immune cells such as activated T cells and NK cells (25–27), and a high neutrophil-to-lymphocyte ratio (NLR) has been reported to be a poor prognostic indicator in various cancers including NSCLC (22, 28, 29). In the context of immunotherapy, a lower baseline NLR correlates with better prognosis in patients with NSCLC treated with ICI therapy (22, 30–34). Additionally, our study and others have shown that successful ICI therapy induces an expansion of the peripheral CX3C chemokine receptor 1 (CX3CR1)^+^ CD8^+^ T cells, which include tumor-specific and tumor-infiltrating T-cell repertoires, in preclinical models and humans (35, 36), and early on-treatment increases in circulating CX3CR1^+^ CD8^+^ T cells are linked to better response and prognosis in patients with NSCLC treated with anti–PD-1/PD-L1 therapy and chemo-immunotherapy (36, 37). However, steroids may affect the number and function of immune cells including neutrophils and T cells (38, 39), and the impact of baseline steroid use on circulating biomarkers in patients with NSCLC remains largely unknown.

Here, we evaluate the impact of baseline steroid use on clinical outcomes and blood-based predictive correlates of response to ICI therapy in patients with NSCLC using independent cohorts from two academic institutions. Our results confirm the poor prognosis associated with baseline steroid use and provide insights into the decreased T-cell effector differentiation and utility of predictive markers by steroids.

## Patients and Methods

### Study design and patients

The study population consisted of patients with naïve or previously treated stage II to IV NSCLC, receiving either anti–PD-1 antibody alone (pembrolizumab or durvalumab), anti–PD-1/CTLA-4 antibodies (nivolumab/ipilimumab), or a combination of chemotherapy and anti–PD-1/PD-L1 antibody (pembrolizumab or atezolizumab). Patients were treated at Roswell Park Comprehensive Cancer Center (RPCCC; *n* = 88) and University of Southern California (USC; Norris Comprehensive Cancer Center or Los Angeles General Medical Center; *n* = 189). This study was approved by the Institutional Review Boards of RPCCC (I 188310) or USC (HS-15-00200) and conducted in accordance with the Declaration of Helsinki.

### Data reporting

The clinical data were retrospectively collected and abstracted by physicians. Because of logistic constraints, no formal power analysis was conducted to predetermine the sample size. The study utilized a convenience sampling approach, with data collected based on availability during the study period. Given the observational nature of the research, randomization and blinding procedures were not implemented. Investigators were not blinded during the study or analysis. We investigated the correlation between biomarker performance during treatment and patient response.

### Assessment of response

Clinical response to immunotherapy or chemo-immunotherapy was assessed as best response per the immune-related RECIST at 6 to 12 weeks as described (37, 40). Responders consisted of patients who had achieved either a complete response or partial response. Nonresponders consisted of patients who had stable disease or progressive disease. The overall response rate (ORR) was calculated as [(complete response + partial response) ÷ number of patients] × 100.

### Blood sample collection and flow cytometry

Blood samples were prospectively collected before treatment and every 3 to 6 weeks at the time of standard-of-care blood draw for 12 weeks at RPCCC. Peripheral blood mononuclear cells (PBMC) were isolated and stored as previously described (36, 37, 40). Fresh or cryopreserved PBMCs were examined for CX3CR1 expression on CD8^+^ T cells as described (36, 37, 40). Briefly, PBMCs were incubated with anti–human IgG (Sigma), followed by staining with a cocktail of antibodies including anti–human CX3CR1 (clone 2A9-1, BioLegend), CD3 (clone UCHT1, BioLegend), CD4 (clone RPA-T4, BD Biosciences), CD8 (clone RPA-T8, BioLegend), CD19 (clone HIB19, BioLegend), CD45 (clone HI30, BD Biosciences), and CD56 (clone HCD56, BioLegend). Flow cytometric data were acquired using LSRFortessa (BD Biosciences) and evaluated with FlowJo software v10.1.5 (FlowJo LLC).

### Mice

Female C57BL/6 mice were purchased from The Jackson Laboratory. All mice were age matched (7–10 weeks old) at the beginning of each experiment and kept under specific pathogen-free conditions and housed in the Laboratory Animal Resources. All animal studies were conducted in accordance with and approved by the Institutional Animal Care and Use Committee at RPCCC.

### Cell lines

The MC38 murine colon adenocarcinoma cell line was a gift from Dr. Weiping Zou (University of Michigan), and it was cultured in RPMI (Gibco) supplemented with 10% FBS (Sigma), 1% Non-Essential Amino Acids (Gibco), 2 mmol/L GlutaMAX-1 (Gibco), 100 U/mL penicillin–streptomycin (Gibco), and 55 μmol/L 2-mercaptoethanol (Gibco). The cells were authenticated by morphology, phenotype, and growth and routinely screened for *Mycoplasma* by PCR and were maintained at 37°C in a humidified atmosphere with 5% CO_2_.

### *In vivo* mouse studies

Female C57BL/6 mice were inoculated with 5 to 8 × 10^5^ MC38 cells per mouse on the right flank by subcutaneous injection on day 0. When tumor volume reached approximately 50 mm^3^, 200 μg of anti–PD-L1 antibody (clone 10F.9G2, Bio X Cell) was administered intraperitoneally every other day. Polyclonal syrian hamster IgG (Bio X Cell) were used as isotype control antibodies. Tumor volumes were calculated by determining the length of short (*l*) and long (*L*) diameters (volume = *l*^2^ × *L*/2). Experimental end points were reached when tumors exceeded 20 mm in diameter or when mice became moribund and showed signs of lateral recumbency, cachexia, lack of response to noxious stimuli, or observable weight loss.

### Statistics

Patient demographics and clinical characteristics were obtained and reported using the mean and range for continuous variables and the frequencies and relative frequencies for categorical variables. The Kaplan–Meier method was used to estimate both overall survival (OS) and progression-free survival (PFS). Right censoring was applied in the survival analyses. For OS, patients were censored at the date of last confirmed contact if they were alive or lost to follow-up. For PFS, censoring occurred at the date of the last tumor assessment for patients who were alive without documented disease progression or who were lost to follow-up. Associations between patient demographics, clinical factors, and survival outcomes were evaluated using Cox proportional hazards regression models. HRs with corresponding 95% confidence intervals (CI) were derived from the model estimates. Variables that were found to be *P* ≤ 0.05 on univariate analysis were further studied in the multivariable analysis. The Fisher exact test was used to determine ORR. CX3CR1 expression in peripheral blood (PB) CD8^+^ T cells was compared using the Mann–Whitney *U* test. The NLR was calculated as absolute neutrophil count divided by absolute lymphocyte count at the initiation of ICI therapy. It was dichotomized using a cutoff value of 5.0, as previously described (30–34). All statistical analyses and graphical representations were performed using GraphPad Prism (v10.3.0).

### Data availability

All data generated and analyzed are available from the corresponding author upon reasonable request.

## Results

### Patient characteristics

We evaluated 277 patients with NSCLC who underwent ICI therapy with treatment initiation between October of 2013 and August of 2023, including 88 patients from RPCCC and 189 from USC. Table 1 summarizes the baseline characteristics of 277 patients. The median age was 66 years, ranging from 30 to 89 years. Of the total cohort, 160 patients (58%) received anti–PD-1/PD-L1 monotherapy whereas 11 patients (4%) had anti–PD-1/CTLA-4 therapy, and 106 patients (38%) were treated with chemo-immunotherapy. The majority of patients had stage IV disease (*n* = 218; 79%), of which 67 patients (24%) had known brain metastases. We identified 21 patients (8%) who were on corticosteroids at the initiation of ICI therapy. The median time of follow-up was 10.4 months for RPCCC patients (range, 0.7–52.0 months) and 6.4 months for USC patients (range, 0.7–88.3 months). Among the 21 patients on steroids, indications included brain metastases (*n* = 17; 80%) or comorbid lung conditions such as chronic obstructive pulmonary disease (*n* = 4; 20%). All 21 patients remained on steroids for at least 12 weeks after starting ICI therapy. We compared the characteristics of patients who received steroids versus those who did not (Table 1). At RPCCC, patients receiving steroids were more likely to have brain metastases (*P* = 0.0001) and to have undergone RT (gamma knife stereotactic radiosurgery; *P* = 0.0021). At USC, patients on steroids were more likely to be treated with chemo-immunotherapy than with immunotherapy alone (*P* = 0.0034).

**Table 1:** Notable patient characteristics from RPCCC and the USC for patients without steroid use compared with patients who received steroids

Characteristic	RPCCC			USC		
	No steroids (*n* = 77)	Steroids (*n* = 11)	*P* value	No steroids (*n* = 179)	Steroids (*n* = 10)	*P* value
Age, years	​	​	​	​	​	​
<65	28	7	0.1058	86	5	>0.9999
≥65	49	4	​	93	5	​
Sex	​	​	​	​	​	​
Female	43	7	0.7508	72	5	0.7426
Male	34	4	​	107	5	​
Race	​	​	​	​	​	​
Caucasian	66	11	​	55	6	​
African American	11	0	​	13	1	​
Asian	0	0	0.3458	57	3	0.1867
Hispanic	0	0	​	45	0	​
Other	0	0	​	9	0	​
ECOG PS	​	​	​	​	​	​
0–1	71	8	0.0810	131	3	0.1559
2–3	6	3	​	21	2	​
History of smoking	​	​	​	​	​	​
Never smoker	6	0	>0.9999	59	1	0.1737
Ever smoker	71	11	​	120	9	​
Histology	​	​	​	​	​	​
Adenocarcinoma	52	9	​	130	9	​
Squamous cell carcinoma	24	1	0.1051	44	0	0.1110
Other	1	1	​	5	1	​
Stage at diagnosis	​	​	​	​	​	​
II–III	18	1	0.4441	39	1	0.6914
IV	59	10	​	140	9	​
Prior lung surgery	21	3	>0.9999	39	1	0.6914
Prior chemotherapy	20	1	0.4484	98	4	0.5171
Prior targeted therapy	5	0	>0.9999	26	2	0.6450
Prior radiation	50	11	0.0021	95	4	0.5225
Known brain metastases	16	9	0.0001	40	2	>0.9999
Drug regimen	​	​	​	​	​	​
Nivolumab or pembrolizumab	47	10	​	101	2	​
Nivolumab and ipilimumab	1	0	​	10	0	​
Carboplatin, paclitaxel, atezolizumab, and bevacizumab	2	0	0.3567	12	0	0.0034
Carboplatin, paclitaxel, and pembrolizumab	9	1	​	14	0	​
Carboplatin, pemetrexed, and pembrolizumab	18	0	​	42	8	​
PD-L1 expression tumor proportion score	​	​	​	​	​	​
<1%	11	3	​	60	4	​
1%–49%	23	1	0.2662	42	3	0.9922
≥50%	43	7	​	41	3	​

Abbreviations: ECOG, Eastern Cooperative Oncology Group; PS, performance status.

### The use of corticosteroids at the initiation of ICI therapy correlates with decreased ORR and worse prognosis in patients with NSCLC

**Figure 1:**
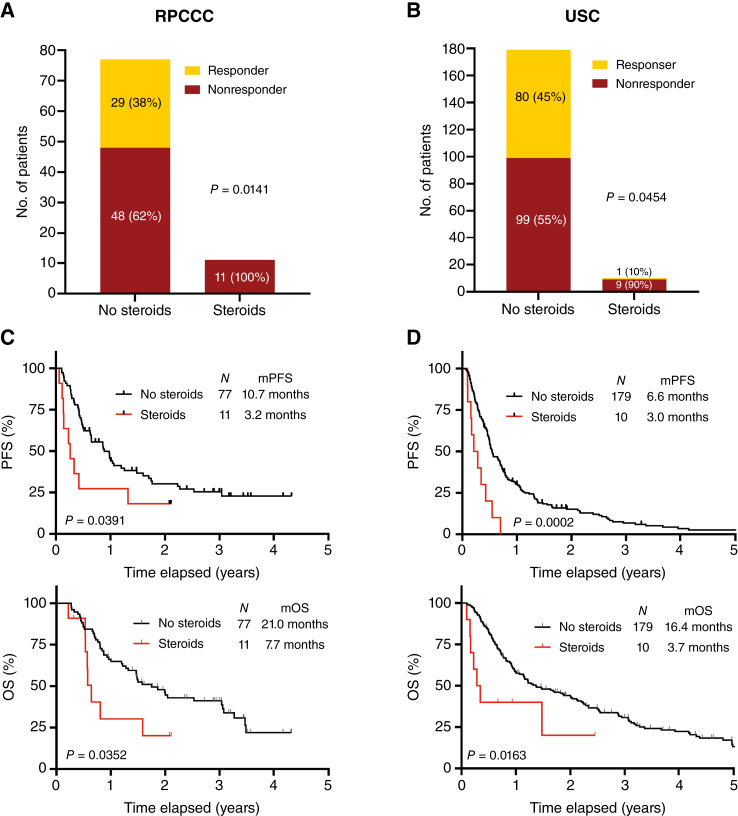
The use of corticosteroids at the initiation of ICI therapy correlates with decreased ORR and worse prognosis in patients with NSCLC. **A–D,** ORR, PFS, and OS of patients at RPCCC (**A** and **C**) and USC (**B** and **D**) treated with ICI therapy on the basis of baseline steroid use. The ORR was analyzed by the Fisher exact test. *P* values for PFF and OS were calculated by a log-rank (Mantel–Cox) test. mOS, median OS; mPFS, median PFS.

We first evaluated the impact of baseline steroid use on ICI therapy outcomes in 277 patients. At RPCCC, among 77 patients who were not on steroids at the start of ICI therapy, 29 (38%) were responders, whereas 48 (62%) were classified as nonresponders (Fig. 1A). At USC, of the 179 patients not on steroids, 80 (45%) were responders and 99 (55%) were nonresponders (Fig. 1B). Baseline corticosteroid use correlated with a decreased ORR in both cohorts (RPCCC: *P* = 0.0141; USC: *P* = 0.0454). Furthermore, patients who did not receive steroids had substantially longer PFS and OS compared with those who received steroids (for RPCCC, median PFS: 10.7 vs. 3.2 months; *P* = 0.0391 and median OS: 21.0 vs. 7.7 months; *P* = 0.0352; Fig. 1C; for USC, median PFS: 6.6 vs. 3.0 months; *P* = 0.0002 and median OS: 16.4 vs. 3.7 months; *P* = 0.0163; Fig. 1D).

### Baseline steroid use is a significant independent risk factor for disease progression and mortality in patients with NSCLC undergoing ICI therapy

To better understand the multiple factors that could potentially influence patient outcomes, univariate and multivariate analyses were performed. In the univariate analysis, baseline steroid use was associated with worse PFS and OS compared with no steroid use (for RPCCC: PFS HR = 2.090; 95% CI, 0.96–4.05; *P* = 0.0423 and OS HR = 2.224; 95% CI, 0.96–4.51; *P* = 0.0397; for USC: PFS HR = 3.300; 95% CI, 1.61–6.03; *P* = 0.0003 and OS HR = 2.484; 95% CI, 1.05–4.96; *P* = 0.020; Table 2). At both institutions, squamous cell carcinoma histology was associated with worse outcomes compared with adenocarcinoma (for RPCCC: PFS HR = 2.264; 95% CI, 1.29–3.89; *P* = 0.004 and OS HR = 1.915; 95% CI, 1.08–3.31; *P* = 0.022; for USC: OS HR = 1.626; 95% CI, 1.10–2.36; *P* = 0.012). At RPCCC, prior lung surgery correlated with longer OS compared with no surgery (OS HR = 0.495; 95% CI, 0.25–0.91; *P* = 0.032) whereas it did not correlate with PFS. At USC, Hispanic race tended to have worse PFS (PFS HR = 1.500; 95% CI, 0.99–2.25; *P* = 0.050) but not OS. Never-smokers showed significantly worse PFS (PFS HR = 0.654; 95% CI, 0.48–0.91; *P* = 0.010) but no difference in OS. Patients with stage III disease had longer OS compared with those with stage IV disease (OS HR = 1.711; 95% CI, 1.12–2.72; *P* = 0.017).

**Table 2:** Univariate analyses of PFS and OS in the RPCCC (left) and USC (right) cohorts

​	RPCCC						USC					
	Progression			Mortality			Progression			Mortality		
	HR	95% CI	*P* value	HR	95% CI	*P* value	HR	95% CI	*P* value	HR	95% CI	*P* value
Age	1.006	0.98–1.03	0.621	1.010	0.98–1.04	0.445	0.999	0.98–1.01	0.785	1.008	0.99–1.03	0.372
Sex	​	​	​	​	​	​	​	​	​	​	​	​
Female (ref)	​	​	​	​	​	​	​	​	​	​	​	​
Male	1.033	0.62–1.69	0.897	1.341	0.79–2.26	0.272	0.796	0.59–1.08	0.138	1.082	0.77–1.52	0.644
Race	​						​					
Caucasian (ref)	​	​	​	​	​	​	​	​	​	​	​	​
African American	​	​	​	​	​	​	1.208	0.63–2.15	0.543	0.985	0.48–1.84	0.964
Asian	0.460	0.16–1.04	0.100	0.604	0.21–1.37	0.282	1.095	0.75–1.60	0.637	0.787	0.52–1.20	0.263
Hispanic	​	​	​	​	​	​	1.500	0.99–2.25	0.050	1.110	0.72–1.70	0.635
Other	​	​	​	​	​	​	1.106	0.48–2.20	0.791	0.889	0.34–1.93	0.788
ECOG PS	​						​					
0–1 (ref)	​	​	​	​	​	​	​	​	​	​	​	​
2–3	0.925	0.38–1.64	0.846	1.349	0.55–2.81	0.463	1.195	0.73–1.86	0.452	1.255	0.75–1.99	0.357
Smoking	​						​					
Never smoker (ref)	​	​	​	​	​	​	​	​	​	​	​	​
Ever smoker	0.632	0.30–1.64	0.285	0.453	0.21–1.18	0.068	0.654	0.48–0.91	0.010	0.998	0.71–1.43	0.993
Histology	​						​					
Adenocarcinoma (ref)	​	​	​	​	​	​	​	​	​	​	​	​
Squamous cell	2.264	1.29–3.89	0.004	1.915	1.08–3.31	0.022	1.183	0.81–1.68	0.363	1.626	1.10–2.36	0.012
Other	0.816	0.05–3.77	0.841	1.073	0.06–4.99	0.945	2.486	0.97–5.23	0.031	3.578	1.24–8.14	0.007
Stage	​						​					
III (ref)	​	​	​	​	​	​	​	​	​	​	​	​
IV	1.078	0.61–2.03	0.804	1.487	0.79–3.04	0.242	1.437	1.00–2.12	0.058	1.711	1.12–2.72	0.017
Prior lung surgery	​						​					
No (ref)	​	​	​	​	​	​	​	​	​	​	​	​
Yes	0.804	0.45–1.37	0.441	0.495	0.25–0.91	0.032	0.737	0.50–1.07	0.117	0.925	0.60–1.37	0.708
Prior chemotherapy	​						​					
No (ref)	​	​	​	​	​	​	​	​	​	​	​	​
Yes	1.207	0.68–2.05	0.500	0.946	0.51–1.67	0.854	1.173	0.87–1.59	0.296	0.768	0.58–1.03	0.073
Brain metastases	​						​					
No (ref)	​	​	​	​	​	​	​	​	​	​	​	​
Yes	0.925	0.50–1.61	0.792	1.080	0.57–1.94	0.804	0.760	0.53–1.07	0.131	0.883	0.58–1.30	0.544
PD-L1 expression	​						​					
0%–1% (ref)	​	​	​	​	​	​	​	​	​	​	​	​
1%–49%	0.974	0.44–2.29	0.949	0.918	0.40–2.23	0.844	0.930	0.62–1.37	0.716	0.988	0.63–1.53	0.956
≥50%	1.027	0.52–2.27	0.944	1.150	0.58–2.55	0.709	0.958	0.63–1.43	0.835	1.095	0.69–1.71	0.694
Steroid use	​						​					
No (ref)	​	​	​	​	​	​	​	​	​	​	​	​
Yes	2.090	0.96–4.05	0.042	2.224	0.96–4.51	0.040	3.300	1.61–6.03	0.0003	2.484	1.05–4.96	0.020

Abbreviations: ECOG, Eastern Cooperative Oncology Group; PS, performance status; ref, reference.

For the multivariate analysis, all variables with significant *P* values ≤ 0.05 were included (Table 3). At RPCCC, the multivariate analysis for PFS confirmed the significance of steroid use and histology, consistent with the univariate analysis. Notably, the HR for steroids increased and the *P* value showed a greater degree of significance (PFS HR = 2.670; 95% CI, 1.20–5.35; *P* = 0.009). A similar trend was observed in the multivariate analysis for OS, with an increased HR and a significant *P* value for steroids (OS HR = 2.443; 95% CI, 1.03–5.20; *P* = 0.029). Histology remained significant in the multivariate PFS and OS analyses whereas prior lung surgery was not significant. At USC, similar results were observed, with higher HRs for steroid use in PFS and OS (PFS HR = 4.339; 95% CI, 2.06–8.23; *P* < 0.0001 and OS HR = 3.161; 95% CI, 1.32–6.42; *P* = 0.004). Never-smoking remained significant in the multivariate PFS analysis whereas Hispanic race and histology did not. In the multivariate OS analysis, histology and stage remained significant.

**Table 3:** Multivariate Cox regression analyses of demographic and clinical characteristics that proved significant in the univariate analysis in Table 2

​	RPCCC					
​	Progression			Mortality		
	HR	95% CI	*P* value	HR	95% CI	*P* value
Histology	​	​	​	​	​	​
Adenocarcinoma (ref)	​	​	​	​	​	​
Squamous cell carcinoma	2.586	1.45–4.54	0.001	1.957	1.08–3.50	0.025
Other	0.889	0.05–4.13	0.908	0.855	0.05–4.03	0.878
Prior lung surgery	​	​	​	​	​	​
No (ref)	​	​	​	​	​	​
Yes	​	​	​	0.598	0.30–1.13	0.130
Steroid use	​	​	​	​	​	​
No (ref)	​	​	​	​	​	​
Yes	2.679	1.20–5.35	0.009	2.443	1.03–5.20	0.029

​	USC					
	Progression			Mortality		
	HR	95% CI	*P* value	HR	95% CI	*P* value
Race	​	​	​	​	​	​
Caucasian (ref)	​	​	​	​	​	​
African American	1.334	0.69–2.40	0.362	​	​	​
Asian	1.127	0.75–1.69	0.565	​	​	​
Hispanic	1.498	0.97–2.31	0.067	​	​	​
Other	1.307	0.57–2.62	0.487	​	​	​
Smoking	​	​	​	​	​	​
Never smoker (ref)	​	​	​	​	​	​
Ever smoker	0.639	0.45–0.90	0.011	​	​	​
Histology	​	​	​	​	​	​
Adenocarcinoma (ref)	​	​	​	​	​	​
Squamous cell carcinoma	1.319	0.90–1.90	0.148	1.859	1.25–2.71	0.002
Other	2.787	1.07–6.01	0.018	4.166	1.44–9.51	0.002
Stage	​	​	​	​	​	​
III (ref)	​	​	​	​	​	​
IV	​	​	​	1.924	1.26–3.07	0.004
Steroid use	​	​	​	​	​	​
No (ref)	​	​	​	​	​	​
Yes	4.339	2.06–8.23	<0.0001	3.161	1.32–6.42	0.004

Abbreviation: ref, reference.

### The impact of dose and timing of corticosteroids on clinical outcomes of patients with NSCLC undergoing ICI therapy

Given the demonstrated negative impact of steroid use on progression and mortality, patients on steroids at the initiation of ICI therapy were categorized into medium-dose (>7.5 mg but ≤30 mg prednisone equivalent daily) and high-dose (>30 mg but ≤100 mg prednisone equivalent daily) groups (41). Among the 21 patients on baseline steroids, nine patients (43%; six patients at RPCCC and three patients at USC) received medium doses, and 12 patients (57%; five patients at RPCCC and seven patients at USC) received high doses. In the univariate analysis, high-dose steroids were associated with markedly worse PFS and OS compared with no steroids (for RPCCC: PFS HR = 3.222; 95% CI, 1.11–7.41; *P* = 0.014 and OS HR = 3.693; 95% CI, 1.26–8.63; *P* = 0.007; for USC: PFS HR = 5.450; 95% CI, 2.26–11.16; *P* < 0.0001 and OS HR = 10.48; 95% CI, 3.96–23.11; *P* < 0.0001). Medium-dose steroids, however, did not show a significant difference from no steroids in both PFS and OS (Supplementary Table S1). In the multivariate analysis, high-dose steroids were independently associated with worse prognosis in both cohorts. At RPCCC, high-dose steroid use was linked to worse PFS (HR = 3.915; 95% CI, 1.34–9.20; *P* = 0.005) and OS (HR = 3.857; 95% CI, 1.30–9.28; *P* = 0.006). Similarly, at USC, high-dose steroids were independently associated with worse PFS (HR = 6.010; 95% CI, 2.45–12.63; *P* < 0.0001) and OS (HR = 11.14; 95% CI, 4.15–25.10; *P* < 0.0001; Supplementary Table S2).

A previous study showed that patients who discontinued corticosteroids 1 to 30 days before the initiation of anti–PD-1 therapy had better PFS and OS compared with those who were on corticosteroids at the time of treatment initiation and continued during anti–PD-1 therapy (7). In our analysis, four patients at RPCCC and nine patients at USC discontinued corticosteroids 1 to 30 days before starting ICI therapy. These patients exhibited a better ORR compared with those who were on corticosteroids at treatment initiation and continued during ICI therapy (RPCCC: *P* = 0.0013; USC: *P* = 0.0329; Supplementary Fig. S1). Taken together, our results highlight the multitude of negative effects of baseline and continuous corticosteroid use on ICI therapy efficacy in patients with NSCLC.

### The predictive and prognostic value of a baseline NLR in patients with NSCLC treated with ICI therapy

**Figure 2:**
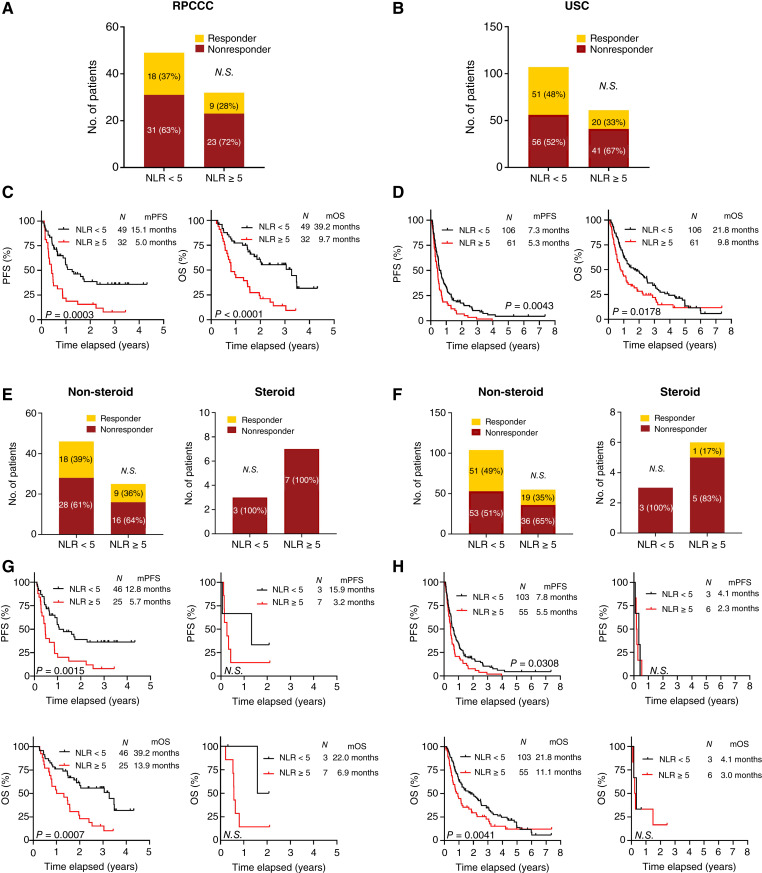
The impact of baseline steroid use on predictive and prognostic values of NLR in patients with NSCLC treated with ICI therapy. **A–H,** ORR, PFS, and OS of patients at RPCCC and USC treated with ICI therapy according to PB-high (≥5) or-low (<5) NLR at baseline. The ORR (**A**, **B**, **E**, and **F**) was analyzed by the Fisher exact test. *P* values for PFF and OS (**C**, **D**, **G**, and **H**) were calculated by a log-rank (Mantel–Cox) test. mOS, median OS; mPFS, median PFS.

Next, we examined the impact of peri-treatment steroid use on PB NLR, which has been reported as a predictive correlate of response in patients with NSCLC undergoing ICI therapy (30–32). Baseline NLR data were available for 81 patients from RPCCC and 171 from USC. In both cohorts, an NLR less than five was associated with longer PFS (RPCCC: *P* = 0.0003; USC: *P* = 0.0043) and OS (RPCCC: *P* < 0.0001; USC: *P* = 0.0178) but was not correlated with response to ICI therapy (Fig. 2A–D). These patients were stratified into two groups—those on steroids and those not on steroids at the initiation of ICI therapy—to evaluate the impact of steroids on the predictive and prognostic value of NLR. A baseline NLR less than five was linked to better prognosis of the non-steroid group but not the steroid group whereas it was not associated with response to ICI therapy in either group (Fig. 2E–H).

### Decreased frequency of circulating CD8^+^ T cells expressing CX3CR1 in patients on corticosteroids

**Figure 3:**
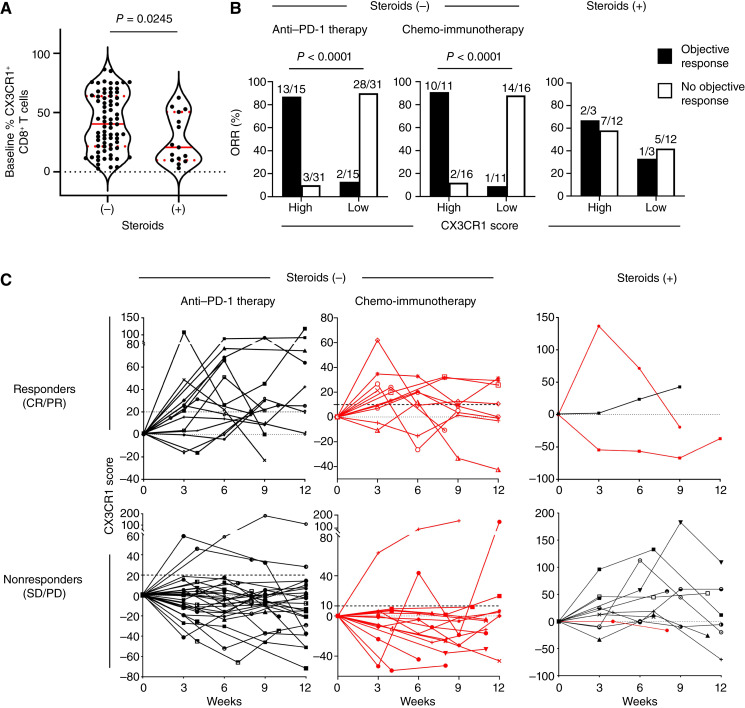
Impact of steroids on CD8^+^ T-cell differentiation and predictive value of the CX3CR1 score in patients with NSCLC treated with ICI therapy. **A,** Violin plot with median (red solid line) and the top and bottom quartiles (red dotted line) showing the frequency of PB CX3CR1^+^ CD8^+^ T cells in patients who were or were not on steroids at baseline. *P* value was calculated by a two-tailed Mann–Whitney *U* test. **B,** ORR for patients who were or were not on steroids at baseline and the maximal percent change of the CX3CR1^+^ subset among PB CD8^+^ T cells from baseline (CX3CR1 score) at 9 weeks. The ORR was analyzed by the Fisher exact test. **C,** CX3CR1 score in responders (CR/PR) and nonresponders (SD/PD) who were or were not on steroids at baseline. The non-steroid group was separated into anti–PD-1 therapy (black line) and chemo-immunotherapy (red line). CR, complete response; PD, progressive disease; PR, partial response; SD, stable disease.

To evaluate the impact of corticosteroids on adaptive immunity, we assessed the expression of chemokine receptor CX3CR1, a marker of T-cell differentiation (42, 43). Given the compelling evidence showing that glucocorticoids suppress T-cell differentiation (38, 39), we analyzed the baseline and on-treatment frequency of PB CX3CR1^+^ CD8^+^ T cells in patients who were on steroids within 30 days of starting ICI therapy. Although the data showed a bimodal distribution, the baseline frequency of PB CX3CR1^+^ CD8^+^ T cells was substantially lower in patients on steroids (*n* = 15) compared with patients not on steroids before treatment (*n* = 72; Fig. 3A). Our recent findings indicate that the percent change of the CX3CR1^+^ subset in PB CD8^+^ T cells from baseline (CX3CR1 score) correlates with response to ICI therapy in which an increase of at least 20% and 10% predicts response to anti–PD-1 therapy and chemo-immunotherapy, respectively (37, 40). In this study, we explored whether peri-treatment corticosteroid use affected early changes in the frequency of PB CX3CR1^+^ CD8^+^ T cells after ICI initiation (on-treatment) and the predictive value of the CX3CR1 score. In patients not using corticosteroids, the maximal CX3CR1 score of at least 20 for anti–PD-1 therapy and of at least 10 for chemo-immunotherapy correlated with the ORR at 9 weeks (anti–PD-1 therapy: *P* < 0.0001; chemo-immunotherapy: *P* < 0.0001) whereas the correlation was not observed in patients on corticosteroids within 30 days of starting ICI therapy (Fig. 3B). Notably, among patients on corticosteroids, the CX3CR1 score indicated that the patient responded to ICI therapy whereas they did not (false positive) in 58.3% (7/12) of cases (Fig. 3B and C). These findings illustrate that the peri-treatment corticosteroid use may impair T-cell differentiation and diminish the predictive accuracy of the CX3CR1 score.

### Continued use of corticosteroids during anti–PD-L1 therapy correlates with decreased survival in a preclinical model

**Figure 4:**
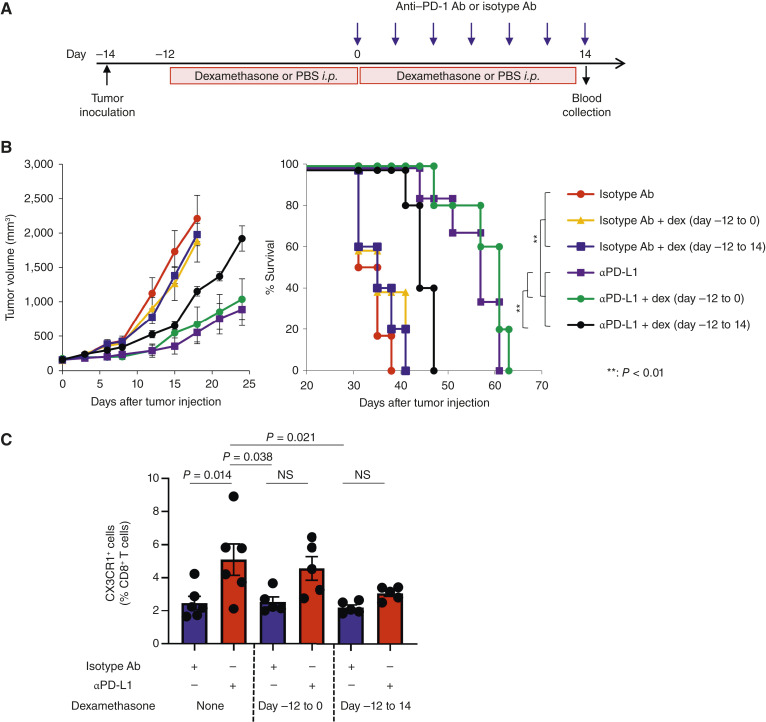
Continued use of corticosteroids during anti–PD-L1 therapy correlates with decreased survival in a preclinical model. **A,** Experimental set-up. Mice bearing MC38 tumors were treated with anti–PD-L1 or isotype antibody. Mice were administered with dexamethasone or PBS intraperitoneally before (day −12 to 0) and/or during (day 0–14) anti–PD-L1 therapy. **B,** Tumor growth curves (mean) and survival curves in MC38 tumor-bearing mice in different treatment groups as indicated. *n* = 5 to 6 mice. **, *P* < 0.01, *P* values were determined by a log-rank (Mantel–Cox) test. **C,** Frequency of the CX3CR1^+^ subset in PB CD8^+^ T cells. PB was collected on day 14. *n* = 5 to 6 mice. *P* values were determined by the Kruskal–Wallis test with Dunn multiple comparisons. Data are presented as mean ± SEM. Ab, antibody; dex, dexamethasone; i.p., intraperitoneal; NS, not significant; αPD-L1, anti–PD-L1.

To gain mechanistic insights on the role of peri-treatment steroids on the efficacy of ICI therapy, we employed a “bedside-to-bench” approach in which we evaluated the impact of pre- and on-treatment steroids on the efficacy of ICI therapy and T-cell differentiation in a preclinical model. We treated mice bearing MC38 colon adenocarcinoma with anti–PD-L1 therapy in the absence or presence of dexamethasone which was continued throughout or discontinued at the time of initiation of anti–PD-L1 therapy (Fig. 4A). We found that pre- and on-treatment steroid administration substantially decreased antitumor efficacy of anti–PD-L1 therapy and survival (Fig. 4B). However, we did not observe the negative impact of dexamethasone when it was discontinued at the time of initiation of anti–PD-L1 therapy. Lastly, we evaluated T-cell differentiation and found that peri-treatment steroid use attenuated the increase of circulating CX3CR1^+^ CD8^+^ T cells during anti–PD-L1 therapy (Fig. 4C).

## Discussion

Glucocorticoids are frequently used in patients with NSCLC, but their impact on those treated with ICI therapy remains elusive. The current study highlights the multitude of negative effects that baseline steroids have on ICI therapy. Patients who received steroids had consistently worse outcomes including lower ORR and shorter PFS and OS. Univariate and multivariate analyses revealed that high-dose steroids correlated with worse outcomes more than any other prognostic factors such as smoking status, ECOG performance, and brain metastases. Patients who discontinued steroids before the induction of ICI therapy had a better response rate compared with those who continued during ICI therapy. The use of steroids was associated with a lower frequency of PB CX3CR1^+^ CD8^+^ T cells, suggesting that steroids may limit full effector differentiation of CD8^+^ T cells. Systemic effects of steroids complicate the interpretation of predictive blood-based biomarkers such as NLR and the CX3CR1 score, reducing their prognostic value.

The majority of patients in our study received steroids for brain metastases and related symptoms similar to prior studies (7, 8, 11–13, 44–46). However, the mere use of steroids was a more influential prognostic factor than the presence of brain metastases as evident in our univariate and multivariate analyses consistent with other studies (9, 12, 13). We found that squamous cell carcinoma histology, advanced staging, and no prior lung surgery were worse prognostic factors for progression or mortality in univariate analysis in either cohort. However, steroid use remained the only substantially higher independent risk factor for progression and mortality in both the RPCCC and USC cohorts on multivariate analysis. These findings underscore the profound impact of steroids on patients treated with ICI therapy.

We found that higher doses of baseline steroids in comparison with lower doses severely affected ICI efficacy and patient outcomes. Our results are in line with previous studies showing that patients who received ≥10 mg of prednisone had substantially lower response rates and worse OS and PFS than those receiving <10 mg of prednisone (7, 8). Furthermore, our study in patients and mice suggested that the timing of steroids might be important in line with a previous study showing that patients who were tapered off steroids had intermediate PFS and OS compared with those who received steroids on the day of ICI initiation (7). Although we recognize that these results must be interpreted with caution given that the number of patients on the medium dose of steroids and those who discontinued steroids was small, these findings provide rationale for future studies to test whether clinical outcomes might be improved by reducing the dose or stopping steroids prior to the initiation of the ICI therapy.

Steroids suppress the immune system and are thus used to treat autoimmune disease and immune-related adverse effects. However, the exact mechanisms underlying immunosuppression in the context of ICI therapy remain unclear. In our study, we observed markedly lower CX3CR1 expression in CD8^+^ T cells at baseline in patients on steroids, consistent with previous studies suggesting the negative impact steroids had on T-cell differentiation (38, 39). In agreement with this, T-cell differentiation was attenuated in mice on steroids before and during anti–PD-L1 therapy and associated with the substantially decreased therapeutic efficacy. This mechanism also affected the utility of the CX3CR1 score as a predictive biomarker; change of the frequency of CX3CR1^+^ CD8^+^ T cells from the baseline did not correlate with response to ICI therapy in patients and tumor-bearing mice on steroids even though steroids were discontinued at the time of steroids initiation. Additionally, steroids are known to influence the frequency and number of peripheral neutrophils and lymphocytes. Although we confirmed that the baseline NLR correlates with prognosis in patients treated with ICI therapy in line with previous studies (22, 28, 29), this was not the case in those on steroids. Taken together, the predictive and prognostic value of circulating immune-related markers should be interpreted with caution in patients on steroids because of their inhibitory effects on a wide variety of immune cells.

Our study has several limitations. The frequency of patients on steroids (8%) was smaller than other previous studies (10.1%–16.4%; refs, 7, 8, 10–13). In contrast, the frequency of brain metastases (an indication of steroids) was relatively higher (24.2%) compared with other research studies (5.8%–26%; refs. 7, 8, 11–13, 44–46). This minor difference might be due to the shared consensus that steroids negatively affect immunotherapy, leading to physicians reducing their use over time, especially given the extended period captured in our study. This likely reflects efforts to reduce steroid use before ICI therapy based on earlier research showing worse outcomes with baseline steroids. The number of patients on a medium dose of steroids (7.5–30 mg prednisone equivalent daily) was small although results were consistent in both cohorts in univariate analysis (Supplementary Table S1). Although our preclinical study suggests that cessation of steroids restored antitumor efficacy of anti–PD-L1 therapy consistent with a better response rate in patients who tapered off steroids than patients who continued steroids, these results need to be interpreted with caution because we only included one mouse model, and the duration of pretreatment steroids might be different in mice and patients. The scope of our study is limited to investigating the impact of steroids within 30 days of initiating ICI therapy, unlike other studies that evaluated steroid use during ICI therapy (44–46). Not all patients in our cohorts had data available to assess the impact of steroids on circulating biomarkers, NLR and CD8^+^ T-cell CX3CR1 expression. Additionally, the USC cohort included a more diverse patient population than the RPCCC cohort, with a higher frequency of Hispanic and Asian patients. Our univariate analysis suggested that Hispanic patients treated with ICI therapy had worse prognosis compared with non-Hispanic Whites although this was not statistically significant in multivariate analysis. Further research with larger cohorts is needed to evaluate the difference in clinical outcomes from ICI therapy between Hispanic patients and non-Hispanic White patients.

In summary, our findings demonstrate that baseline steroid use, particularly at high doses, decreases the efficacy of ICI therapy for patients with NSCLC and correlates with decreased PB CX3CR1^+^ CD8^+^ T cells. Additionally, we found that caution should be taken when interpreting the results from circulating immune-related biomarkers in patients on steroids at the start of ICI therapy.

## Supplementary Material

Table S1 Univariate analyses of PFS and OS in the RPCCC (left) and USC (right) cohorts.

Table S2 Multivariate Cox regression analyses of demographic and clinical characteristics that proved significant in the univariate analysis in Table S1

Supplementary Figure 1 The impact of timing of corticosteroids on response rate in NSCLC patients undergoing ICI therapy.
