# Supplementary material for: Impact of Glucocorticoids on Immune Checkpoint Inhibitor Efficacy and Circulating Biomarkers in Non–Small Cell Lung Cancer Patients
Source: Cancer Res Commun. 2025 Jul 7;5(7):1082–94. doi: 10.1158/2767-9764.CRC-25-0051 (PMC12232904; doi:10.1158/2767-9764.CRC-25-0051)
Supplement: Table S1 — Univariate analyses of PFS and OS in the RPCCC (left) and USC (right) cohorts. [file crc-25-0051_table_s1_suppst1.pdf]

	Roswell Park Comprehensive Cancer Center						University of Southern California					
	Progression			Mortality			Progression			Mortality		
	HR	95% CI	*p*-value	HR	95% CI	*p*-value	HR	95% CI	*p*-value	HR	95% CI	*p*-value
**Age**	1.006	0.98-1.03	0.621	1.010	0.98-1.04	0.445	0.999	0.98-1.01	0.785	1.008	0.99-1.03	0.372
**Sex**												
Female (ref)												
Male	1.033	0.62-1.69	0.897	1.341	0.79-2.26	0.272	0.796	0.59-1.08	0.138	1.082	0.77-1.52	0.644
**Race**												
Caucasian (ref)												
African American							1.208	0.63-2.15	0.543	0.985	0.48-1.84	0.964
Asian	0.460	0.16-1.04	0.100	0.604	0.21-1.37	0.282	1.095	0.75-1.60	0.637	0.787	0.52-1.20	0.263
Hispanic							1.500	0.99-2.25	0.050	1.110	0.72-1.70	0.635
Other							1.106	0.48-2.20	0.791	0.889	0.34-1.93	0.788
**ECOG PS**												
0-1 (ref)												
2-3	0.925	0.38-1.64	0.846	1.349	0.55-2.81	0.463	1.195	0.73-1.86	0.452	1.255	0.75-1.99	0.357
**Smoking**												
Never Smoker (ref)												
Ever Smoker	0.632	0.30-1.64	0.285	0.453	0.21-1.18	0.068	0.654	0.48-0.91	0.010	0.998	0.71-1.43	0.993
**Histology**												
Adenocarcinoma (ref)												
Squamous cell	2.264	1.29-3.89	0.004	1.915	1.08-3.31	0.22	1.183	0.81-1.68	0.363	1.626	1.10-2.36	0.012
Other	0.816	0.05-3.77	0.841	1.073	0.06-4.99	0.945	2.486	0.97-5.23	0.031	3.578	1.24-8.14	0.007
**Stage**												
III (ref)												
IV	1.078	0.61-2.03	0.804	1.487	0.79-3.04	0.242	1.437	1.00-2.12	0.058	1.711	1.12-2.72	0.017
**Prior Lung Surgery**												
No (ref)												
Yes	0.804	0.45-1.37	0.441	0.495	0.25-0.91	0.032	0.737	0.50-1.07	0.117	0.925	0.60-1.37	0.708
**Prior Chemotherapy**												
No (ref)												
Yes	1.207	0.68-2.05	0.500	0.946	0.51-1.67	0.854	1.173	0.87-1.59	0.296	0.768	0.58-1.03	0.073
**Brain Metastases**												
No (ref)												
Yes	0.925	0.50-1.61	0.792	1.080	0.57-1.94	0.804	0.760	0.53-1.07	0.131	0.883	0.58-1.30	0.544
**PD-L1 Expression**												
0-1% (ref)												
1-49%	0.974	0.44-2.29	0.949	0.918	0.40-2.23	0.844	0.930	0.62-1.37	0.716	0.988	0.63-1.53	0.956
≥50%	1.027	0.52-2.27	0.944	1.150	0.58-2.55	0.709	0.958	0.63-1.43	0.835	1.095	0.69-1.71	0.694
**Steroid Use**												
No (ref)												
Medium	1.455	0.44-3.57	0.472	1.344	0.32-3.70	0.622	1.745	0.43-4.63	0.342	0.454	0.03-2.03	0.432
High	3.222	1.11-7.41	0.014	3.693	1.26-8.63	0.007	5.450	2.26-11.2	<.0001	10.48	3.96-23.11	<.0001

Table S1: Univariate analyses of PFS and OS in the RPCCC (left) and USC (right) cohorts. ECOG, Eastern Cooperative Oncology Group
