# Supplementary material for: Impact of Glucocorticoids on Immune Checkpoint Inhibitor Efficacy and Circulating Biomarkers in Non–Small Cell Lung Cancer Patients
Source: Cancer Res Commun. 2025 Jul 7;5(7):1082–94. doi: 10.1158/2767-9764.CRC-25-0051 (PMC12232904; doi:10.1158/2767-9764.CRC-25-0051)
Supplement: Table S2 — Multivariate Cox regression analyses of demographic and clinical characteristics that proved significant in the univariate analysis in Table S1 [file crc-25-0051_table_s2_suppst2.pdf]

	Roswell Park Comprehensive Cancer Center					
	Progression			Mortality		
	Hazard Ratio	95% CI	*p*-value	Hazard Ratio	95% CI	*p*-value
**Histology**						
Adenocarcinoma (ref)						
Squamous Cell Carcinoma	2.550	1.44-4.47	0.001	1.912	1.05-3.41	0.023
Other	0.896	0.05-4.16	0.914	0.958	0.05-4.57	0.967
**Prior Lung Surgery**						
No (ref)						
Yes				0.593	0.29-1.12	0.124
**Steroid Use**						
No (ref)						
Medium	1.898	0.56-4.82	0.229	1.494	0.35-4.32	0.515
High	**3.915**	**1.34-9.20**	**0.005**	**3.857**	**1.30-9.28**	**0.006**

	University of Southern California					
	Progression			Mortality		
	Hazard Ratio	95% CI	*p*-value	Hazard Ratio	95% CI	*p*-value
**Race**						
Caucasian (ref)						
African American	1.274	0.66-2.30	0.445			
Asian	1.086	0.72-1.64	0.692			
Hispanic	1.473	0.95-2.27	0.079			
Other	1.273	0.56-2.55	0.530			
**Smoking**						
Never Smoker (ref)						
Ever Smoker	0.650	0.46-0.92	0.014			
**Histology**						
Adenocarcinoma (ref)						
Squamous Cell Carcinoma	1.306	0.89-1.88	0.163	1.850	1.24-2.70	0.002
Other	2.605	0.99-5.67	0.029	3.941	1.36-9.04	0.004
**Stage**						
III (ref)						
IV				1.801	1.17-2.87	0.010
**Steroid Use**						
No (ref)						
Medium	2.605	0.62-7.29	0.114	0.608	0.03-2.75	0.622
High	**6.010**	**2.45-12.63**	**<0.0001**	**11.14**	**4.15-25.10**	**<0.0001**

Abbreviations: CI, Confidence Interval

Table S2: Multivariate Cox regression analyses of demographic and clinical characteristics that proved significant in the univariate analysis in Table S1.
