# Supplementary material for: Impact of Glucocorticoids on Immune Checkpoint Inhibitor Efficacy and Circulating Biomarkers in Non–Small Cell Lung Cancer Patients
Source: Cancer Res Commun. 2025 Jul 7;5(7):1082–94. doi: 10.1158/2767-9764.CRC-25-0051 (PMC12232904; doi:10.1158/2767-9764.CRC-25-0051)
Supplement: Supplementary Figure 1 — The impact of timing of corticosteroids on response rate in NSCLC patients undergoing ICI therapy. [file crc-25-0051_supplementary_figure_1_suppsf1.pdf]

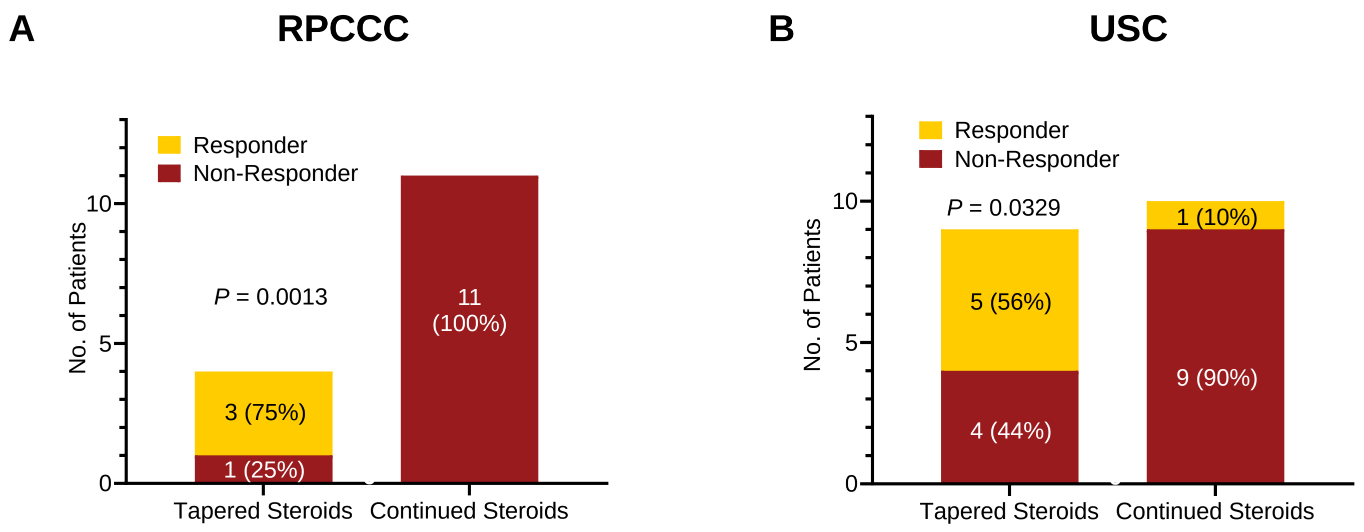

Supplementary Figure 1: The impact of timing of corticosteroids on response rate in NSCLC patients undergoing ICI therapy.

A, B) The impact of tapering steroids on ORR before initiation of ICI therapy for patients at RPCCC (A) and USC (B). ORR was analyzed by Fisher's exact test.
